# Designing highly crystalline multifunctional multicolor-luminescence nanosystem for tracking breast cancer heterogeneity

**DOI:** 10.1039/c8na00089a

**Published:** 2018-11-26

**Authors:** Avijit Pramanik, Salma Begum, Chris Rightsell, Kaelin Gates, Qinku Zhang, Stacy Jones, Ye Gao, Vikram Ruppa-Kasani, Rimika Banerjee, Jayanti Shukla, Ashley Ignatius, Dhiraj Sardar, Fengxiang. X. Han, Paresh Chandra Ray

**Affiliations:** Department of Chemistry and Biochemistry, Jackson State University Jackson MS USA paresh.c.ray@jsums.edu + 16019793674; Department of Physics and Astronomy, University of Texas at San Antonio San Antonio Texas 78249 USA

## Abstract

Breast tumor heterogeneity was responsible for the death of ∼40 000 women in 2017 in the USA. Triple-negative breast cancers (TNBCs) are very aggressive, and this is the only subgroup of breast cancers that still lacks effective therapeutics. As a result, the early-stage detection of TNBCs is vital and will have huge significance in clinical practice. Driven by this need, we here report the design of highly crystalline antibody-conjugated multifunctional multicolor-luminescence nanosystems derived from the naturally available popular tropical fruits mangoes and prunes, which have the ability to detect breast cancer heterogeneity *via* the selective separation and accurate identification of TNBC and HER-2(+) or ER/PR(+) breast cancer cells selectively and simultaneously. The detailed synthesis and characterization of the multifunctional multicolor nanosystems derived from tropical fruits have been reported. Experimental results show that by changing the fruits multicolor-luminescence carbon dots (LCDs) can be developed, which is mainly due to the formation of highly crystalline nanodots with different heavy metal dopants and is also due to the presence of different types of surface functional group. Experimental data that are presented show that the multifunctional multicolor nanoprobe can be used for the highly selective and simultaneous capture of targeted TNBC and HER-2(+) or ER(+) breast cancer cells, and the capture efficiency can be as high as 98%. Reported data indicate that multicolor fluorescence imaging can be used for mapping heterogeneous breast cancer cells simultaneously and can distinguish targeted TNBC from non-targeted HER-2(+) or ER/PR(+) breast cancer. Our finding suggests the excellent potential of the design of multicolor nanosystems derived from natural fruits for detecting cancer heterogeneity in clinical practice.

## Abstract 2

Fruit based nanosystem can identify triple negative breast cancer cells.

## Graphical abstract

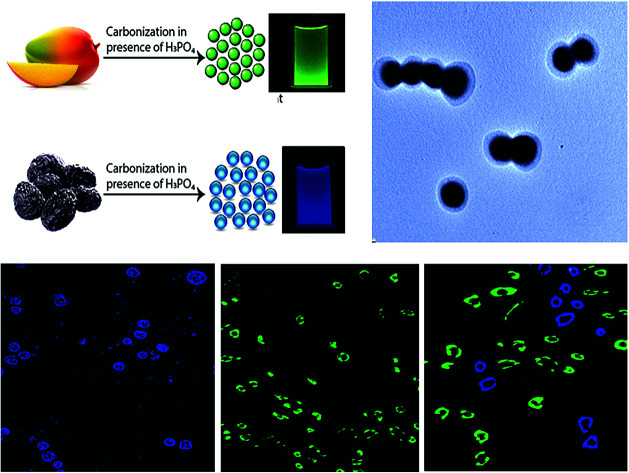

## Introduction

Although breast cancer has been known since 3000 B.C.E., it is the second leading cause of cancer deaths in women.^1–3^ It is now well documented that breast cancer heterogeneity is the main obstacle to the effective treatment of breast cancer, which was responsible for the deaths of 40 610 women and 460 men in 2017 in the USA alone.^1–3^

**Scheme 1:**
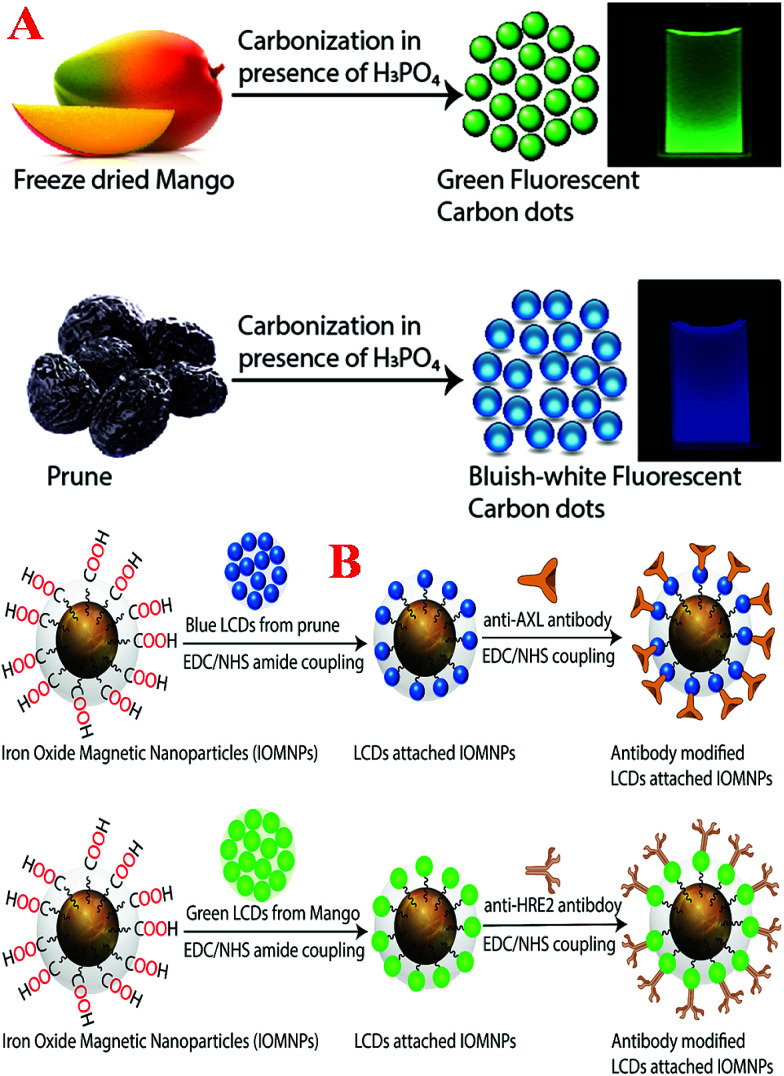
(A) Fruit-based synthetic route we used for the development of blue and green fluorescent carbon dots. (B) Synthetic procedure we used for the development of antibody-conjugated blue and green fluorescent magnetic LCDs.

Even now, personal clinical decisions regarding targeted breast cancer treatment rely primarily on the identification of three markers, namely, two types of hormone receptor, estrogen receptor (ER) and progesterone receptor (PR), and human epidermal growth factor receptor 2 (HER-2).^4–19^ Because triple negative breast cancer (TNBC) lacks hormone receptors and HER-2, currently there is no targeted therapy for TNBC in clinical practice, and as a result patients with TNBC suffer from very poor therapeutic outcomes.^4–10^ TNBC is well known to be a highly aggressive form of cancer, and as a result the standard techniques used in clinics such as mammograms, magnetic resonance imaging (MRI) and ultrasound usually detect TNBCs at later stages.^1–15^ The above facts clearly indicate that mapping breast cancer heterogeneity is one of the highest priorities of current breast cancer research for improving the treatment of TNBC. Driven by this need, we here report a facile approach for the design of highly crystalline antibody-conjugated multifunctional multicolor-luminescence nanosystems derived from the naturally available popular tropical fruits mangoes and prunes, as shown in Schemes 1A and B, which have the ability to detect breast cancer heterogeneity *via* the selective separation and accurate identification of TNBC and HER-2(+) or ER/PR(+) breast cancer cells from a mixture.

Luminescent carbon dots (LCDs) are a new class of bioimaging nanomaterials, of which the surface contains multiple oxygen-containing moieties.^20–30^ Because LCDs exhibit remarkably bright photoluminescence due to quantum confinement effects and also exhibit very high biocompatibility, photostability and aqueous solubility,^30–40^ LCDs can be highly promising candidates for applications in daily life.^41–49^ The photoluminescence wavelength of LCDs can be varied by varying their size, shape and surface states.^20–30^ Because the surface states of LCDs are critical to their wavelength-dependent photoemission properties,^30–40^ we report a green synthesis route for the development of blue- and green-colored LCDs by controlling the contents of nitrogen (N), sulfur (S), phosphorus (P) and other minerals using prune and mango fruits.^50,51^ Prunes are dried fruits of the plum *Prunus domestica* L., which contain phytochemicals, sorbitol, volatile compounds and minerals such as phosphorus, boron, copper, manganese, potassium, iron and retinol.^50^ The mango, which has the scientific name *Mangifera indica* L., contains more than 270 volatile compounds, including ethyl butanoate and 4-hydroxy-2,5-dimethyl-3(2*H*)-furanone, numerous phytochemicals and minerals such as phosphorus, zinc, selenium, potassium and magnesium.^51^ By changing the fruits, we produced LCDs with different colors, which were mainly due to the presence of different surface functional groups, which introduced new energy levels for electron transitions with comparable intensities. Similarly, with the change of fruits, doping with different heavy metals occurred, which also introduced new energy levels for electron transitions. For the selective separation and imaging of TNBC cells, we developed multifunctional magnetic-fluorescent LCDs by developing LCDs to which magnetic nanoparticles were attached. In our design, the magnetic properties of the multifunctional LCDs were used for the selective separation of each type of breast cancer cell, namely, HER-2(+), ER/PR(+) or TNBC. Furthermore, the multicolor-fluorescence LCDs were used to visualize different types of breast cancer cell for detection *via* multicolor fluorescence imaging to provide an accurate diagnosis.

## Results and discussion

### Design of blue fluorescent LCDs from prunes

Blue fluorescent LCDs were synthesized by the hydrothermal treatment of prunes using H_3_PO_4_.

**Fig. 1:**
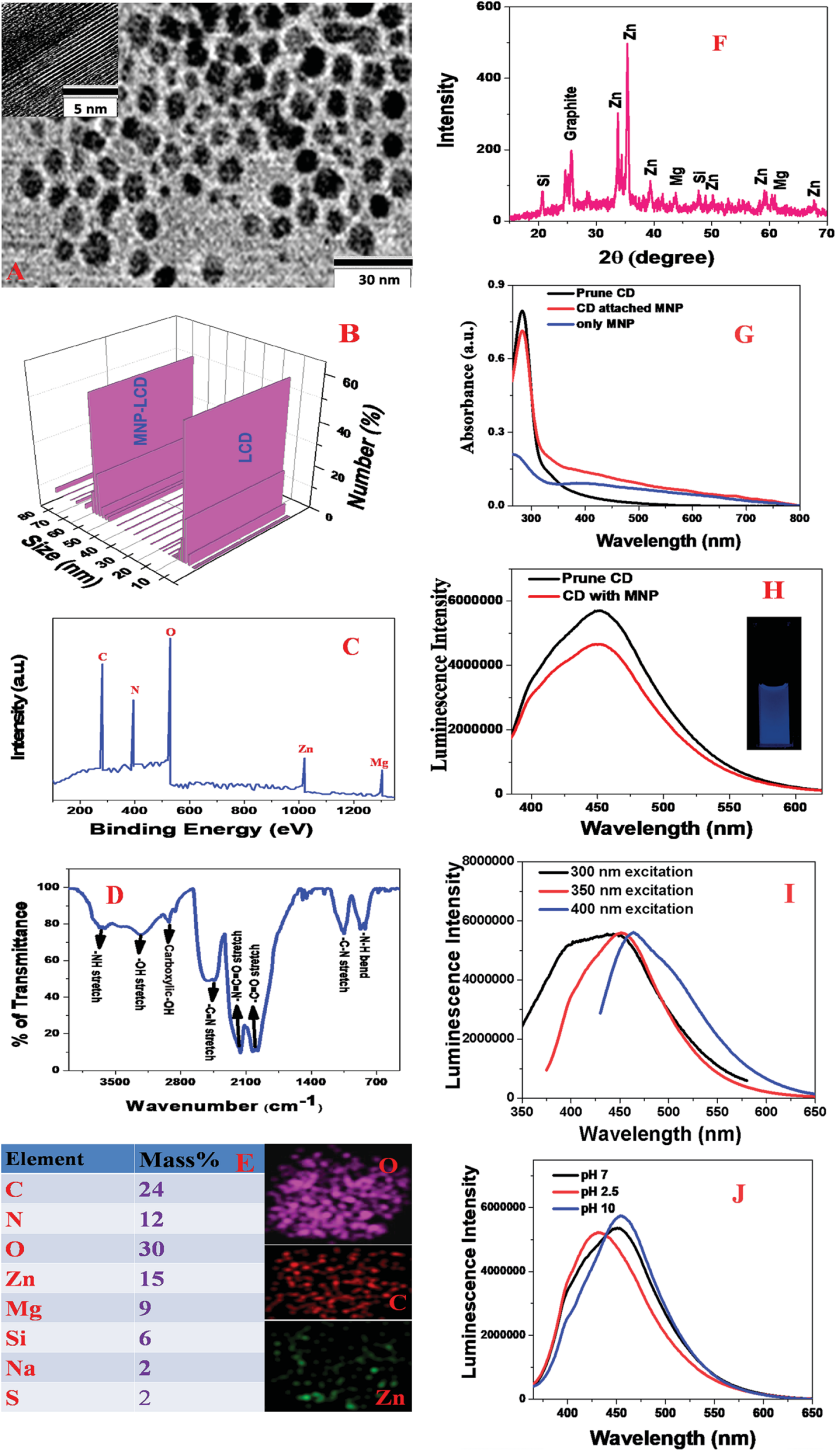
(A) TEM image of freshly prepared blue luminescent CDs from prunes. Inset: HR-TEM image of a single particle showing well-resolved lattice fringes with an interplanar spacing of 0.38 nm, which are due to graphitic carbon. (B) DLS data showing a histogram of the size distributions of the freshly prepared blue luminescent CDs (LCDs) from prunes and LCDs attached to magnetic nanoparticles. (C) XPS spectrum of freshly prepared blue luminescent CDs from prunes showing peaks due to carbon, N, O, Zn and Mg at 284.8, 398, 532.8, 1024, and 1303 eV, respectively. (D) FTIR spectrum of freshly prepared LCDs from prunes showing the presence of different types of surface functional group, such as –NH, –OH, C=O, C=N, –N=C=O, *etc.* (E) EDX elemental mapping of freshly prepared LCDs from prunes showing the presence of N, C, O, Zn, Mg, *etc.* (F) X-ray diffraction (XRD) pattern of freshly prepared blue luminescent CDs from prunes, which also confirms the presence of graphitic carbon, Zn, Mg and Si. (G) UV-vis absorption spectra of freshly prepared CDs from prunes, magnetic nanoparticles and CDs attached to magnetic nanoparticles. (H) Emission spectra of freshly prepared CDs from prunes and CDs attached to magnetic nanoparticles at an excitation wavelength of 380 nm. The inset photograph shows the blue-colored emission from freshly prepared CDs from prunes in the presence of UV light. (I) Excitation wavelength-dependent emission spectra of freshly prepared CDs from prunes. (J) Emission spectra of freshly prepared CDs from prunes at different pH values.

The experimental details are described in the Experimental section. For this purpose, dried prunes were dissolved in water and H_3_PO_4_. After sonication for 5 min, the solution was put in an autoclave and heated at 180 °C for 2 h. The product was then neutralized with NaOH. Finally, the product was dialyzed against water for 72 h and then filtered. The blue fluorescent carbon dots were further purified by dialysis and then stored at 4 °C. After the synthesis, we purified the particles using silica column chromatography. Finally, the prune-based LCDs were characterized by high-resolution tunneling electron microscopy (HR-TEM), energy-dispersive X-ray (EDX) spectroscopy, powder X-ray diffraction (XRD), X-ray photoelectron spectroscopy (XPS), infrared (IR) spectroscopy, fluorescence spectroscopy and dynamic light scattering (DLS) measurements,^15–19,29,46,49^ as reported in Fig. 1A–J. Fig. 1A shows a TEM image of freshly prepared LCDs from prunes, which indicates that the size of the prune-based LCDs was 8 ± 2 nm. Fig. 1B and Table 1 report the DLS data, which also indicate that the average size of freshly prepared LCDs from prunes was about 7 ± 3 nm, which matches the TEM data quite well. To understand and characterize the lattice structure, we also performed high-resolution TEM (HR-TEM) on freshly prepared LCDs from prunes. The HR-TEM image of a single particle of freshly prepared LCDs from prunes in the inset of Fig. 1A shows well-resolved lattice fringes with an interplanar spacing of 0.38 nm, which are due to graphitic carbon. The observed spacing is slightly larger than that of bulk graphite, which is 0.334 nm. This is due to the presence of functional groups and nitrogen, Zn, Mg and other dopant atoms, which increased the basal plane spacing. Fig. 1C shows the XPS spectrum of freshly prepared blue luminescent CDs from prunes, which indicates the presence of carbon, N, O, Zn and Mg by the presence of peaks due to C_1s_ (25.0%), O_1s_ (10%), N_1s_ (31%), Mg_1s_ (10%), and Zn_2p_ (15%). EDX elemental mapping of freshly prepared LCDs from prunes, as reported in Fig. 1E, indicates the presence of N, C, O, K, Mg, Zn, *etc.* The EDX elemental analysis data match the XPS analysis data very well. High-resolution XPS analysis indicates the presence of several different types of C-binding site, namely, –CN, C=O, C–C and COO.

**Table 1:** Size distribution of blue-colored fluorescent LCDs from prunes, acid-functionalized magnetic nanoparticles and multifunctional prune-based LCDs

Nanoparticle description	Size measured by DLS	Size measured by TEM
Prune-based LCDs	7 ± 3 nm	8 ± 2 nm
Acid-functionalized magnetic nanoparticles	45 ± 10 nm	40 ± 5 nm
Multifunctional prune-based LCDs	58 ± 12 nm	55 ± 10 nm

Fig. 1D shows the Fourier transform infrared (FTIR) spectrum of freshly prepared LCDs from prunes, which indicates the presence of several different types of surface functional group such as –NH, –OH, C=O, C=N, –N=C=O, *etc.* The reported FTIR spectrum exhibits a clear peak at ∼3650 cm^−1^, which corresponds to –NH stretching vibrations. Similarly, we also observed a vibration band at ∼3280 cm^−1^, which corresponds to the absorption band due to O–H stretching. The other FTIR bands observed at ∼3020 cm^−1^, ∼2450 cm^−1^, ∼2270 cm^−1^, ∼1980 cm^−1^, ∼1100 cm^−1^ and ∼780 cm^−1^ correspond to carboxylic acid –OH stretching, C≡N stretching, –N=C=O stretching, acid C=O stretching, amine C–N stretching, and –NH bending vibrations, respectively. Fig. 1F shows the powder X-ray diffraction (XRD) pattern of freshly prepared blue luminescent CDs from prunes, which also confirms the presence of graphitic carbon, Zn, Mg and Si, as all the peaks match the JCPDS XRD peaks for ZnO, MgO, SiO_2_ and graphitic carbon. To understand the luminescence behavior, we also recorded the luminescence spectrum at an excitation wavelength of 380 nm. Fig. 1G shows the absorption spectrum of freshly prepared CDs from prunes, which exhibits a strong absorption peak at 282 nm. The observed peak can be attributed to π → π* transitions in C=C bonds and n → π* transitions in C=O bonds. As shown in Fig. 1H, the emission spectrum of freshly prepared LCDs from prunes at an excitation wavelength of 380 nm displays a broad emission from the prune-based LCDs with an emission maximum (*λ*_max_ of emission) around 450 nm. The photograph in the inset of Fig. 1H shows that freshly prepared LCDs from prunes exhibited blue fluorescence when excited at 380 nm with UV light. Using quinine sulfate as a standard (QY = 54%), we determined that the photoluminescence quantum yield of freshly prepared blue luminescent LCDs from prunes was 0.36 at an excitation wavelength of 380 nm. Fig. 1I shows that the photoluminescence spectrum of freshly prepared CDs from prunes can be changed by varying the excitation energy. Although the exact origin of the excitation-dependent single-photon luminescence from freshly prepared CDs from prunes is not known, it may be due to ground-state heterogeneity due to their polydispersity. In addition, there is the possibility of multiple discrete electronic states owing to the presence of different types of aggregate. Fig. 1J shows the luminescence spectra of freshly prepared CDs from prunes at various pH values, which indicate that there were slight changes in intensity as well as in the wavelength of the luminescence maximum as the pH was varied from acidic to basic.

### Design of green fluorescent LCDs from mangoes

Green fluorescent LCDs were synthesized by the hydrothermal treatment of mangoes using H_3_PO_4_. The experimental details are described in the experimental section. In this case, we used a very similar experimental procedure to that used for prunes. For this purpose, ripe mangoes were dissolved in water and H_3_PO_4_. After sonication for 5 min, the solution was put in an autoclave and heated at 200 °C for 2 h. The product was then neutralized with NaOH.

**Fig. 2:**
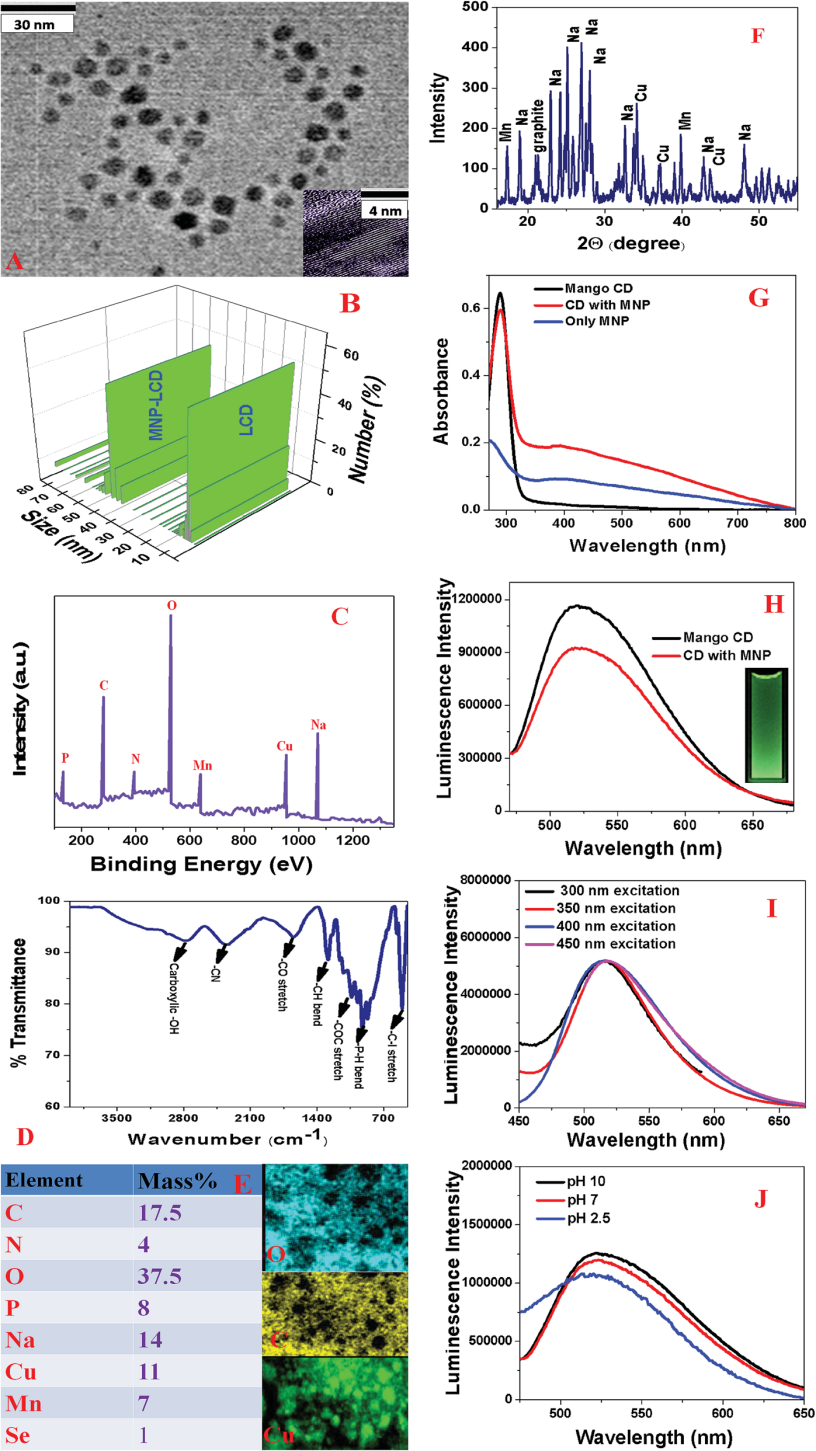
(A) TEM image of freshly prepared green luminescent CDs from mangoes. Inset: HR-TEM image of a single particle showing well-resolved lattice fringes with an interplanar spacing of 0.36 nm, which are due to graphitic carbon. (B) DLS data showing a histogram of the size distributions of freshly prepared green luminescent CDs from mangoes and LCDs attached to magnetic nanoparticles. (C) XPS spectrum of freshly prepared blue luminescent CDs from mangoes showing peaks due to P, C, N, O, Mn, Cu and Na at 128.5, 284.8, 398, 530, 638.7, 933 and 1071 eV, respectively. (D) FTIR spectrum of freshly prepared LCDs from mangoes showing the presence of different types of surface functional group, such as carboxylic –OH, C=O, C=N, –P–H, –C–I, *etc.* (E) EDX elemental mapping of freshly prepared LCDs from mangoes showing the presence of P, C, O, Se, Cu, Mn, *etc.* (F) X-ray diffraction (XRD) pattern of freshly prepared blue luminescent CDs from mangoes, which also confirms the presence of graphitic carbon, Na, Cu and Mn. (G) UV-vis absorption spectra of freshly prepared CDs from mangoes, magnetic nanoparticles and CDs attached to magnetic nanoparticles. (H) Emission spectra of freshly prepared CDs from mangoes and CDs attached to magnetic nanoparticles at an excitation wavelength of 380 nm. The inset photograph shows the green-colored emission from freshly prepared CDs from mangoes in the presence of UV light. (I) Excitation wavelength-dependent emission spectra of freshly prepared CDs from mangoes. (J) Emission spectra of freshly prepared CDs from mangoes at different pH values.

Finally, the product was dialyzed against water for 72 h and then filtered. The green fluorescent carbon dots were further purified by dialysis and then stored at 4 °C. After the synthesis, we purified the particles using silica column chromatography. Finally, the mango-based LCDs with green emissions were characterized by HR-TEM, EDX, FTIR, and fluorescence spectroscopy and DLS measurements, as reported in Fig. 2A–J. Fig. 2A shows a TEM image of freshly prepared LCDs from mangoes, which indicates that the size of the mango-based LCDs was 6 ± 2 nm. Fig. 2B and Table 2 report the DLS data, which also indicate that the average size of freshly prepared LCDs from mangoes was about 5 ± 2 nm, which matches the TEM data quite well. The HR-TEM image of a single particle of freshly prepared LCDs from mangoes in the inset of Fig. 2A shows well-resolved lattice fringes with an interplanar spacing of 0.36 nm, which is similar to that of the (020) diffraction facets of graphitic carbon. The observed spacing is slightly larger than that of bulk graphite, which is 0.334 nm. This is due to the presence of functional groups and nitrogen, P, Cu, Mn and other dopant atoms, which increased the basal plane spacing. Fig. 2C shows the XPS spectrum of freshly prepared green luminescent CDs from mangoes, which indicates the presence of P, C, N, O, Cu and Mn by the presence of peaks due to P_2p_ (8.0%), C_1s_ (19%), O_1s_ (36%), N_1s_ (6%), Na_1s_ (12%), Mn_2p_ (6.0%) and Cu_2p_ (12%). EDX elemental mapping of freshly prepared LCDs from mangoes, as reported in Fig. 2E, indicates the presence of P, N, C, O, Na, Mn, Cu, *etc.* The EDX elemental analysis data closely match the XPS analysis data. High-resolution XPS analysis indicates the presence of several different types of C-binding site, namely, –CN, C=O, C–C, and COO. Fig. 2D shows the FTIR spectrum of freshly prepared LCDs from mangoes, which shows the presence of several different types of surface functional group, such as –PH, C=O, C=N, –C–I, carboxylic –OH, *etc.*Fig. 2F shows the powder X-ray diffraction (XRD) pattern of freshly prepared green luminescent CDs, which also confirms the presence of graphitic carbon, Na, Cu and Mg, as all the peaks match the JCPDS XRD peaks for sodium phosphate, MgO, CuO and graphitic carbon.

**Table 2:** Size distribution of green-colored fluorescent LCDs from mangoes, acid-functionalized magnetic nanoparticles and multifunctional mango-based LCDs

Nanoparticle description	Size measured by DLS	Size measured by TEM
Mango-based LCDs	5 ± 2 nm	4 ± 2 nm
Acid-functionalized magnetic nanoparticles	42 ± 8 nm	40 ± 5 nm
Multifunctional mango-based LCDs	48 ± 10 nm	48 ± 8 nm

Fig. 2G shows the absorption spectrum of freshly prepared CDs from mangoes, which displays a strong absorption peak at 292 nm. The observed peak can be attributed to π → π* transitions in C=C bonds and n → π* transitions in C=O bonds. Fig. 2H shows the emission spectrum of freshly prepared LCDs from mangoes at an excitation wavelength of 380 nm, which displays a broad emission from the mango-based LCDs with an emission maximum (*λ*_max_ of emission) around 530 nm. The photograph in the inset of Fig. 2H shows that freshly prepared LCDs from mangoes exhibited green fluorescence when excited at 380 nm with UV light. Using quinine sulfate as a standard (QY = 54%), we determined that the quantum yield of green fluorescent LCDs from mangoes was 0.61 at an excitation wavelength of 380 nm. Fig. 2I shows the excitation wavelength-dependent photoluminescence spectra of freshly prepared CDs from mangoes, which indicate that the luminescence spectrum was unchanged when we varied the excitation wavelength from 300 nm to 450 nm. Fig. 2J shows the luminescence spectra of freshly prepared CDs from mangoes at various pH values, which indicate that there were slight changes in intensity as the pH was varied from acidic to basic.

### Design of antibody-conjugated blue/green fluorescent multifunctional magnetic fruit-based LCD nanosystems

To detect breast cancer heterogeneity *via* the selective separation and accurate identification of TNBC and HER-2(+) or ER/PR(+) breast cancer cells from blood, we have developed antibody-conjugated multifunctional multicolor-fluorescence magnetic LCD-based nanosystems.

**Fig. 3:**
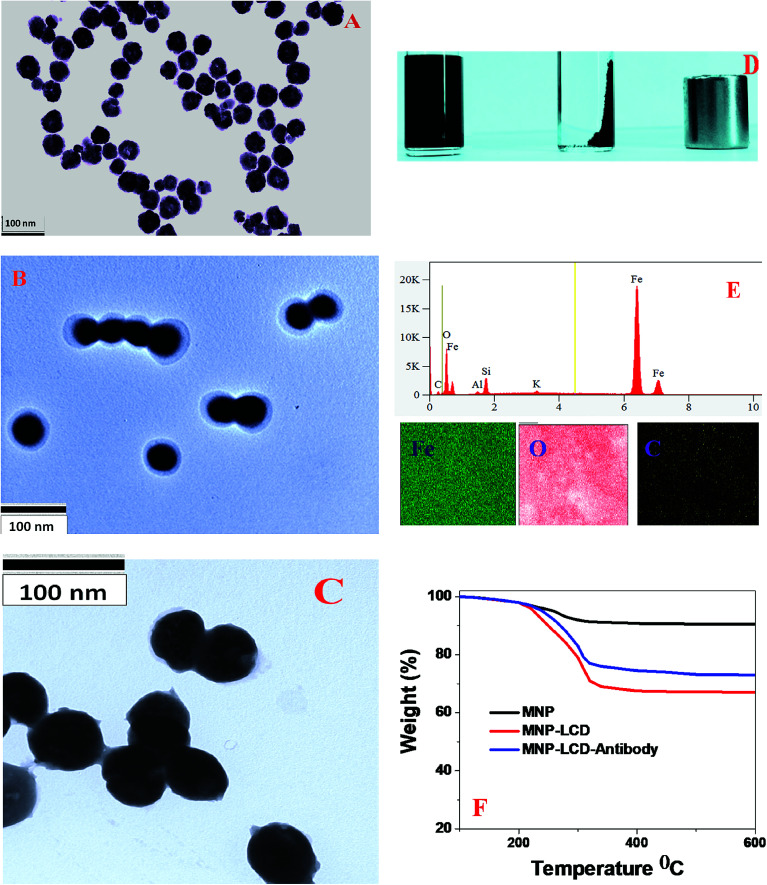
(A) TEM image of freshly prepared acid-functionalized magnetic nanoparticles. (B) TEM image of freshly prepared green fluorescent LCDs attached to a multifunctional magnetic nanosystem. (C) TEM image of freshly prepared blue fluorescent LCDs attached to a multifunctional magnetic nanosystem. (D) Photograph showing highly paramagnetic green fluorescent LCDs attached to a multifunctional nanosystem, which can be separated very easily using a small bar magnet. (E) EDX elemental mapping of freshly prepared green fluorescent LCDs attached to a multifunctional magnetic nanosystem showing the presence of Fe, C, O, Mg, *etc.* (F) Thermogravimetric analysis (TGA) profiles for freshly prepared magnetic nanoparticles, blue fluorescent LCDs and blue fluorescent LCDs attached to a multifunctional magnetic nanosystem.

For the development of a multifunctional multicolor-fluorescence nanoprobe, at first we synthesized carboxylic acid-functionalized magnetic nanoparticles using a co-precipitation method from ferric chloride and 1,6-hexanedioic acid, as we reported previously, as shown in Scheme 1B.^15,16,30^ After that, the black precipitate comprising magnetic Fe_3_O_4_ nanoparticles was separated from the supernatant using a neodymium magnet. In the next step, the acid-functionalized magnetic nanoparticles were characterized by HR-TEM and DLS. The HR-TEM image in Fig. 3A shows that the acid-functionalized magnetic nanoparticles had an average size of about 40 ± 5 nm, which matches the data from DLS measurements, as reported in Tables 1 and 2, quite well. To determine the superparamagnetic properties of the acid-functionalized magnetic nanoparticles, we used a SQUID magnetometer,^17–19,29,46,49^ which indicated that the specific saturation magnetization was 36.2 emu g^−1^. As shown in Scheme 1B, in the next step we carried out EDC-mediated esterification for the development of acid-functionalized magnetic nanoparticles attached to blue/green fluorescent LCDs using a reported method that we have described previously.^17–29^ After that, the ester-coupled blue/green fluorescent LCDs attached to multifunctional magnetic nanoparticles were separated by a magnet, as shown in Fig. 3D. After the magnetic separation, the multifunctional nanosystems were washed several times with water to remove excess LCDs. As shown in Fig. 3B, an HR-TEM image indicates that the size of our green fluorescent LCDs attached to a magnetic nanosystem was 48 ± 8 nm, which matches the results of DLS measurements, as reported in Table 2 and Fig. 2B, quite well. Similarly, as shown in Fig. 3C, an HR-TEM image indicates that the size of our blue fluorescent LCDs attached to a magnetic nanosystem was 55 ± 10 nm, which matches the results of DLS measurements, as reported in Table 1 and Fig. 1B, quite well. We also measured the zeta potentials of magnetic nanoparticles and green luminescent CDs attached to magnetic nanoparticles separately, which indicated that the zeta potential of magnetic nanoparticles peaked at 1.84 ± 0.65 mV, whereas the zeta potential of green luminescent CD-coated magnetic nanoparticles peaked at −1.52 ± 0.43 mV. Fig. 3E shows the EDX data for green fluorescent LCDs attached to magnetic nanoprobes, which clearly show the presence of Fe, C, O, Mg, K, *etc.* To determine the loading amounts of LCDs, we performed thermogravimetric analysis (TGA), as shown in Fig. 3F. From the TGA data we estimated that the weight percentage of blue fluorescent LCDs in the fluorescent magnetic composite was about 14 ± 3%. Similarly, from TGA experiments we estimated that the weight percentage of green fluorescent LCDs in the fluorescent magnetic composite was about 19 ± 3%. Fig. 1G and 2G show the absorption spectra of LCDs attached to magnetic nanoparticles, which exhibit peaks for LCDs as well as for magnetic nanoparticles. Fig. 1H shows the luminescence spectrum of blue fluorescent LCDs attached to magnetic nanoprobes, which indicates that fluorescence quenching of 13 ± 2% occurred after modification with magnetic nanoparticles. Similarly, Fig. 2H shows the luminescence spectrum of green fluorescent LCDs attached to magnetic nanoprobes, which indicates that fluorescence quenching of 16 ± 2% occurred after modification with magnetic nanoparticles. The measurements of superparamagnetic properties indicate that the specific saturation magnetization was 30.2 emu g^−1^ for the blue fluorescent LCDs attached to magnetic nanoprobes and 27.8 emu g^−1^ for the green fluorescent LCDs attached to magnetic nanoprobes.

Finally, for the selective separation and accurate identification of TNBC and HER-2(+) or ER/PR(+) breast cancer cells from blood, we developed antibody-conjugated multifunctional multicolor-fluorescence magnetic LCD-based nanosystems. For this purpose, we developed amine-conjugated polyethylene glycol (NH_2_-PEG) attached to a blue/green fluorescent magnetic LCD-based nanosystem, and then a TNBC-targeted anti-AXL antibody was attached to a green fluorescent magnetic LCD-based nanosystem by our previously reported method.^14,15,30^ Similarly, for the selective separation and imaging of HER-2(+) SK-BR-3 cells, we developed an anti-HER-2 antibody attached to a blue fluorescent magnetic LCD-based nanosystem using the above procedure. On the other hand, for the selective separation and imaging of estrogen receptor (ER)(+) MCF-7 cells, an anti-ERa antibody attached to a blue fluorescent magnetic LCD-based nanosystem was developed. We also measured the zeta potentials of magnetic nanoparticles, blue luminescent CDs attached to magnetic nanoparticles and antibody-conjugated blue luminescent CDs attached to magnetic nanoparticles separately. The experimental measurements indicated that the zeta potential of magnetic nanoparticles peaked at 1.84 ± 0.65 mV, whereas the zeta potential of blue luminescent CD-coated magnetic nanoparticles peaked at −1.98 ± 0.63 mV. On the other hand, the zeta potential of the antibody-conjugated nanoplatform peaked at −3.25 ± 0.81 mV.

To determine the loading amounts of the antibodies, we performed thermogravimetric analysis, as shown in Fig. 3F. From the TGA data we estimated that the weight percentage of the antibodies in the fluorescent magnetic composite was about 4.5 ± 1%. Similarly, from TGA experiments we estimated that the weight percentage of green fluorescent LCDs in the fluorescent magnetic composite was about 5.8 ± 1%.

### Determining the possible toxicity and photostability of the blue/green fluorescent magnetic LCD-based nanosystems

**Fig. 4:**
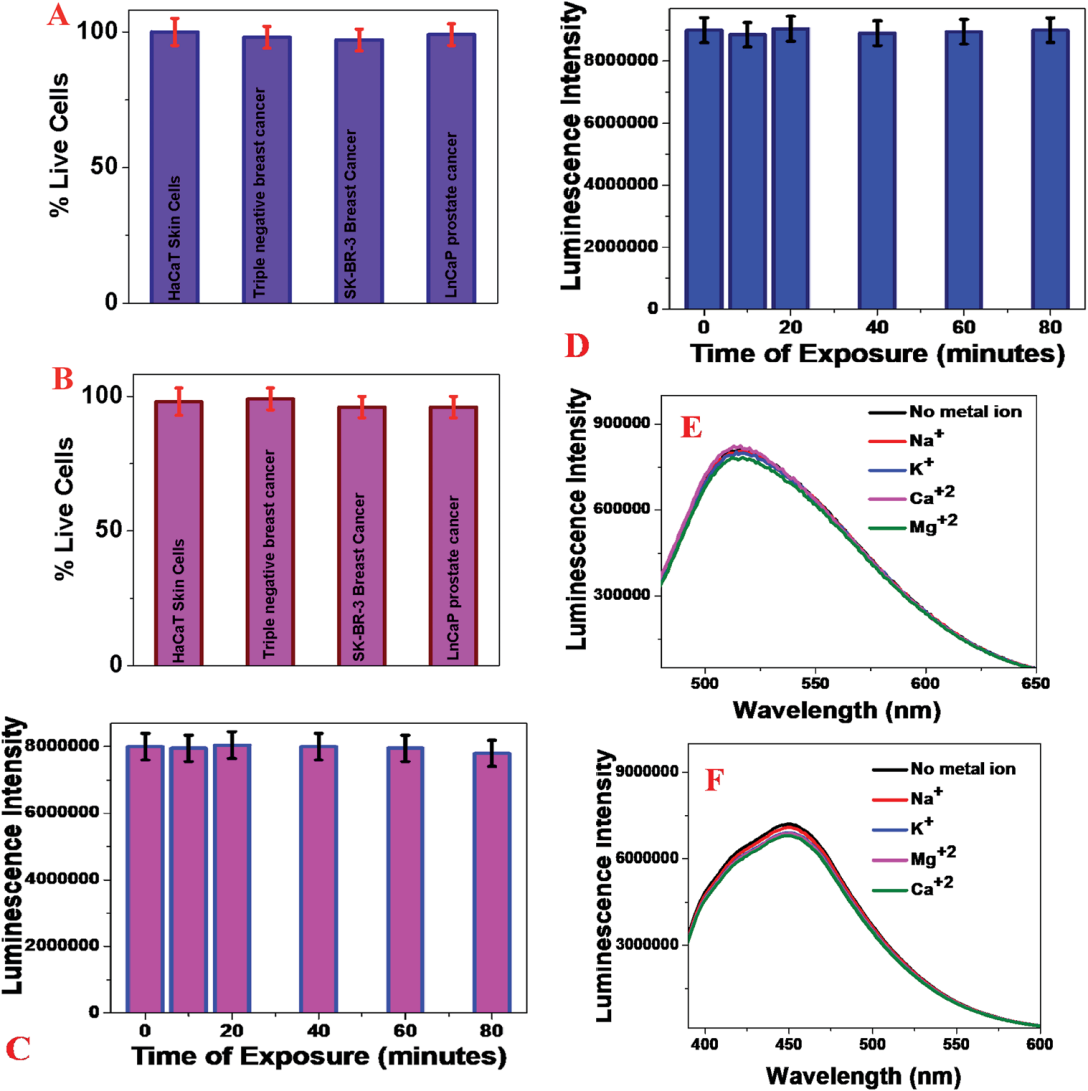
(A) Viability of different breast cancer, prostate cancer and normal skin cells treated with the blue fluorescent magnetic LCD-based nanosystem. The error bars represent the standard deviation (*n* = 3). (B) Viability of different breast cancer, prostate cancer, and normal skin cells treated with the green fluorescent magnetic LCD-based nanosystem. The error bars represent the standard deviation (*n* = 3). (C) Fluorescence intensity of anti-AXL antibody attached to a green fluorescent magnetic LCD-based nanosystem as a function of time. The error bars represent the standard deviation (*n* = 3). (D) Fluorescence intensity of anti-HER-2 antibody attached to a blue fluorescent magnetic LCD-based nanosystem as a function of time. The error bars represent the standard deviation (*n* = 3). (E) Plot showing the influence of metal ions on the luminescence spectrum of green LCDs attached to magnetic nanoparticles. (F) Plot showing the influence of metal ions on the luminescence spectrum of blue LCDs attached to magnetic nanoparticles.

To determine the possible toxicity of our blue/green fluorescent magnetic LCD-based nanosystems, we incubated an antibody attached to a blue/green fluorescent magnetic LCD-based nanosystem with 1.2 × 10^5^ cells per mL normal HaCaT skin cells, HER-2(+) SK-BR-3 cells, MDA-MB-231 TNBC cells and LNCaP human prostate cancer cells separately for 24 h. After that, the numbers of live breast cancer, prostate cancer and normal skin cells were measured using an MTT test.^16–19,29,46,49^Fig. 4A shows the excellent biocompatibility of the blue fluorescent magnetic LCD-based nanosystem for breast cancer, prostate cancer and normal skin cells, as more than 98% of cells were alive after incubation for one day. Similarly, Fig. 4B shows the excellent biocompatibility of the green fluorescent magnetic LCD-based nanosystem for breast cancer, prostate cancer and normal skin cells, as more than 97% of cells were alive after incubation for one day.

Next, to determine the photostability of our developed blue/green fluorescent magnetic LCD-based nanosystems, time-dependent luminescence experiments were performed at an excitation wavelength of 380 nm for an exposure time of 80 min. Fig. 4C shows the excellent photostability of the blue fluorescent magnetic LCD-based nanosystem, of which the luminescence intensity changed by less than 8% even after exposure to light for 80 min. Similarly, Fig. 4D shows the excellent photostability of the green fluorescent magnetic LCD-based nanosystem, of which the luminescence intensity changed by less than 6% even after exposure to light for 80 min. Because blood contains several metal ions that may influence the luminescence spectra of CDs, we performed several luminescence experiments on LCDs attached to magnetic nanoparticles in the presence of different metal ions. As shown in Fig. 4E and F, we did not observe any significant changes in the luminescence intensity of green and blue luminescent LCDs attached to magnetic nanoparticles in the presence of Na^+^, K^+^, Mg^2+^ and Ca^2+^.

### Selective capture and identification of TNBC cells from infected blood and cell mixtures using an anti-AXL antibody attached to a green fluorescent magnetic LCD-based nanosystem

**Fig. 5:**
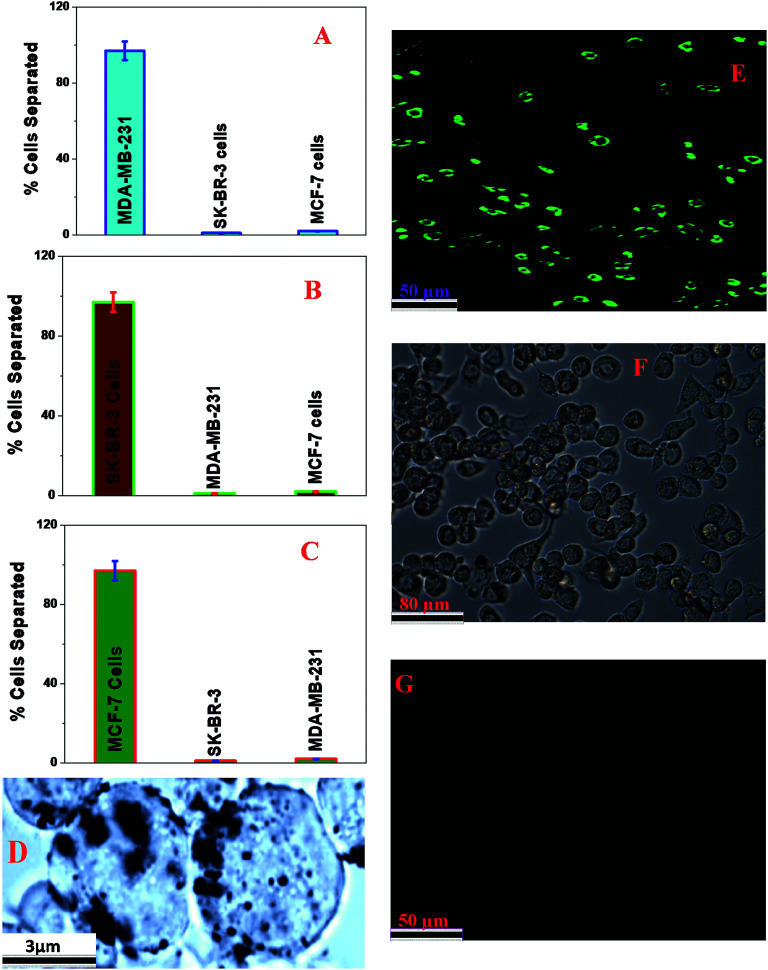
(A) Percentages of HER-2(−) ER(−) MDA-MB-231 cells, HER-2(+) SK-BR-3 cells and ER(+) MCF-7 cells captured by an anti-AXL antibody attached to a green fluorescent magnetic LCD-based nanosystem. The error bars represent the standard deviation (*n* = 3). (B) Percentages of HER-2(−) ER(−) MDA-MB-231 cells, HER-2(+) SK-BR-3 cells and ER(+) MCF-7 cells captured by an anti-HER-2 antibody attached to a blue fluorescent magnetic LCD-based nanosystem. The error bars represent the standard deviation (*n* = 3). (C) Percentages of HER-2(−) ER(−) MDA-MB-231 cells, HER-2(+) SK-BR-3 cells and ER(+) MCF-7 cells captured by an anti-ERa antibody attached to a blue fluorescent magnetic LCD-based nanosystem. The error bars represent the standard deviation (*n* = 3). (D) TEM image showing an anti-AXL antibody attached to a green fluorescent magnetic LCD-based nanosystem on the surface of MDA-MB-231 cells after magnetic separation. (E) Single-photon luminescence image showing MDA-MB-231 cells captured by an anti-AXL antibody attached to a green fluorescent magnetic LCD-based nanosystem. (F) Bright-field image of HER-2(+) SK-BR-3 cells, ER(+) MCF-7 cells, peripheral blood mononuclear cells and rabbit blood cells, which did not bind to an anti-AXL antibody attached to a green fluorescent magnetic LCD-based nanosystem. (G) Single-photon fluorescence image of supernatant indicating that HER-2(+) SK-BR-3 cells, ER(+) MCF-7 cells, peripheral blood mononuclear cells and rabbit blood cells did not bind to an anti-AXL antibody attached to a green fluorescent magnetic LCD-based nanosystem.

To determine whether an anti-AXL antibody attached to a green fluorescent magnetic LCD-based nanosystem can be used for the selective capture and identification of TNBC cells from infected blood and cell mixtures, 10^5^ cells per mL of HER-2(−) ER(−) MDA-MB-231 cells, 10^5^ cells per mL of HER-2(+) SK-BR-3 cells and 10^5^ cells per mL of ER(+) MCF-7 cells were incubated with 10 mL citrated whole rabbit blood. In the next step, we added 10^6^ cells per mL peripheral blood mononuclear cells (PBMCs) to the mixture, which was gently shaken for more than 90 min. After that, different concentrations of an anti-AXL antibody attached to a green fluorescent magnetic LCD-based nanosystem were added to the infected blood sample and mixed continuously for 40 min. After that, the targeted cells bound to the anti-AXL antibody attached to the green fluorescent magnetic LCD-based nanosystem were separated using a bar magnet. In the next step, the targeted cells bound to the anti-AXL antibody attached to the green fluorescent magnetic LCD-based nanosystem were characterized using an enzyme-linked ELISA kit, TEM and fluorescence mapping analysis, as shown in Fig. 5. As shown in Fig. 5A, ELISA experimental data show that the capture efficiency for TNBC cells was greater than 97% when we used the anti-AXL antibody attached to a green fluorescent magnetic LCD-based nanosystem. On the other hand, the capture efficiency for HER-2(+) SK-BR-3 cells and ER(+) MCF-7 cells was less than 3% when we used the anti-AXL antibody attached to a green fluorescent magnetic LCD-based nanosystem. Fig. 5D shows a TEM image of magnetically separated cells, which shows that the anti-AXL antibody attached to a green fluorescent magnetic LCD-based nanosystem was on the surface of the MDA-MB-231 cells that were separated by the magnet.

Fig. 5E shows a green single-photon luminescence image of magnetically captured cells, which indicates that MDA-MB-231 cells were captured by the anti-AXL antibody attached to a green fluorescent magnetic LCD-based nanosystem. Our reported data show that the anti-AXL antibody attached to a green fluorescent magnetic LCD-based nanosystem can be used for imaging TNBC cells *via* a very bright green emission after magnetic separation. The bright-field image shown in Fig. 5F shows that HER-2(+) SK-BR-3 cells, ER(+) MCF-7 cells, peripheral blood mononuclear cells and rabbit blood cells did not bind to the anti-AXL antibody attached to a green fluorescent magnetic LCD-based nanosystem. Similarly, a single-photon fluorescence image of the supernatant indicates that HER-2(+) SK-BR-3 cells, ER(+) MCF-7 cells, peripheral blood mononuclear cells and rabbit blood cells did not bind to the anti-AXL antibody attached to a green fluorescent magnetic LCD-based nanosystem. As a result, we did not observe any luminescence image from the supernatant after magnetic separation. All the reported data clearly indicate that the anti-AXL antibody attached to a green fluorescent magnetic LCD-based nanosystem is highly specific for TNBC cells and can be used for the targeted separation and imaging of MDA-MB-231 and TNBC cells.

### Selective capture and identification of HER-2(+) SK-BR-3 breast cancer cells from infected blood and cell mixtures using an anti-HER-2 antibody attached to a blue fluorescent magnetic LCD-based nanosystem

**Fig. 6:**
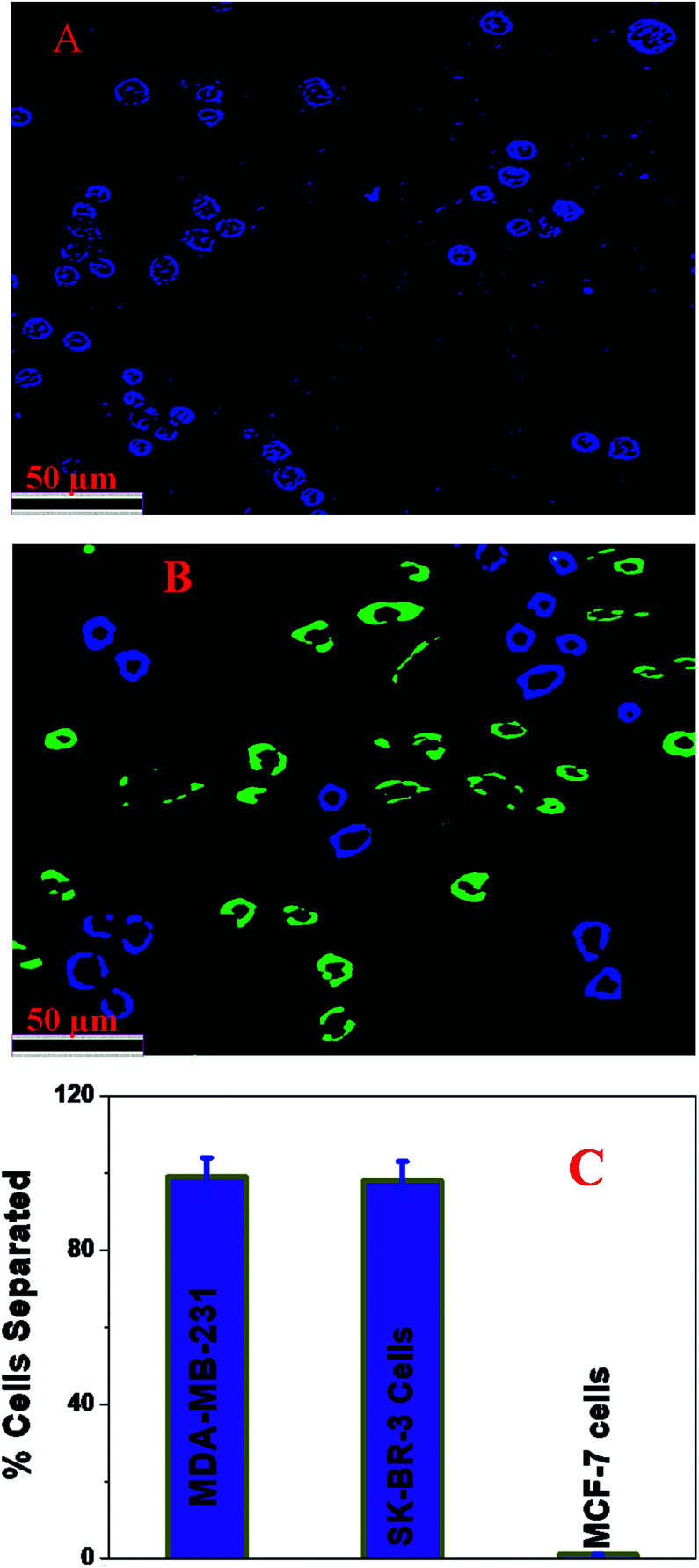
(A) Single-photon luminescence image showing HER-2(+) SK-BR-3 cells captured by an anti-HER-2 antibody attached to a blue fluorescent magnetic LCD-based nanosystem. (B) Multicolor fluorescence image showing that a mixture of an anti-AXL antibody attached to a green fluorescent magnetic LCD-based nanosystem and an anti-HER-2 antibody attached to a blue fluorescent magnetic LCD-based nanosystem was capable of capturing MDA-MB-231 TNBC cells and HER-2(+) SK-BR-3 cells simultaneously from spiked blood. (C) Percentages of HER-2(−) ER(−) MDA-MB-231 cells, HER-2(+) SK-BR-3 cells and ER(+) MCF-7 cells captured by a mixture of an anti-AXL antibody attached to a green fluorescent magnetic LCD-based nanosystem and an anti-HER-2 antibody attached to a blue fluorescent magnetic LCD-based nanosystem. The error bars represent the standard deviation (*n* = 3).

Next, to determine whether an anti-HER-2 antibody attached to a blue fluorescent magnetic LCD-based nanosystem can be used for the selective capture and identification of HER-2(+) breast cancer cells from infected blood and cell mixtures, 10^5^ cells per mL of HER-2(−) ER(−) MDA-MB-231 cells, 10^5^ cells per mL of HER-2(+) SK-BR-3 cells and 10^5^ cells per mL of ER(+) MCF-7 cells were incubated with 10 mL citrated whole rabbit blood. In the next step, we added 10^6^ cells per mL of peripheral blood mononuclear cells (PBMCs) to the mixture and subjected it to gentle shaking for more than 90 min. After that, different concentrations of an anti-HER-2 antibody attached to a blue fluorescent magnetic LCD-based nanosystem were added to the infected blood sample and mixed continuously for 40 min. After that, the targeted cells bound to the anti-HER-2 antibody attached to the blue fluorescent magnetic LCD-based nanosystem were separated using a bar magnet. In the next step, the targeted cells bound to the anti-HER-2 antibody attached to the green fluorescent magnetic LCD-based nanosystem were characterized using an ELISA kit and fluorescence mapping analysis, as shown in Fig. 5 and 6.

As shown in Fig. 5B, ELISA experimental data show that the capture efficiency for HER-2(+) SK-BR-3 cells was greater than 98% when we used an anti-HER-2 antibody attached to a blue fluorescent magnetic LCD-based nanosystem. On the other hand, the capture efficiency for HER-2(−) and ER(−) MDA-MB-231 and ER(+) MCF-7 cells was less than 4% when we used an anti-HER-2 antibody attached to a blue fluorescent magnetic LCD-based nanosystem. Fig. 6A shows a blue single-photon luminescence image of magnetically captured cells, which indicates that HER-2(+) SK-BR-3 cells were captured by the anti-HER-2 antibody attached to a blue fluorescent magnetic LCD-based nanosystem. Our reported data show that the anti-HER-2 antibody attached to a blue fluorescent magnetic LCD-based nanosystem can be used for imaging HER-2(+) cancer cells *via* a very bright green emission after magnetic separation. All the reported data clearly indicate that the anti-HER-2 antibody attached to a blue fluorescent magnetic LCD-based nanosystem is highly specific for HER-2(+) SK-BR-3 cells and can be used for the targeted separation and imaging of HER-2(+) SK-BR-3 cells.

### Selective capture and identification of ER(+) MCF-7 breast cancer cells from infected blood and cell mixtures using an anti-ERa antibody attached to a magnetic LCD-based nanosystem

Next, to determine whether an anti-ERa antibody attached to a magnetic LCD-based nanosystem can be used for the selective capture and identification of ER(+) breast cancer cells from infected blood and cell mixtures, 10^5^ cells per mL of HER-2(−) ER(−) MDA-MB-231 cells, 10^5^ cells per mL of HER-2(+) SK-BR-3 cells and 10^5^ cells per mL of ER(+) MCF-7 cells were incubated with 10 mL citrated whole rabbit blood. In the next step, we added 10^6^ cells per mL peripheral blood mononuclear cells (PBMCs) to the mixture and subjected it to gentle shaking for more than 90 min. After that, different concentrations of an anti-ERa antibody attached to a magnetic LCD-based nanosystem were added to the infected blood sample and mixed continuously for 40 min. After that, the targeted cells bound to the anti-ERa antibody attached to the magnetic LCD-based nanosystem were separated using a bar magnet. In the next step, the targeted cells bound to the anti-ERa antibody attached to the magnetic LCD-based nanosystem were characterized using an ELISA kit, as shown in Fig. 5. As shown in Fig. 5C, ELISA experimental data show that the capture efficiency for ER(+) MCF-7 cells was greater than 98% when we used the anti-ERa antibody attached to a magnetic LCD-based nanosystem. On the other hand, the capture efficiency for HER-2(−) and ER(−) MDA-MB-231 and ER(−) SK-BR-3 cells was less than 3% when we used the anti-ERa antibody attached to a magnetic LCD-based nanosystem. All the reported data clearly indicate that the anti-ERa antibody attached to a magnetic LCD-based nanosystem is highly specific for ER(+) MCF-7 cells and can be used for the targeted separation and imaging of ER(+) MCF-7 cells.

### Simultaneous capture and identification of MDA-MB-231 TNBC cells and HER-2(+) SK-BR-3 breast cancer cells from infected blood and cell mixtures

Next, to determine whether a mixture of an anti-HER-2 antibody attached to a blue fluorescent magnetic LCD-based nanosystem and an anti-AXL antibody attached to a green fluorescent magnetic LCD-based nanosystem can be used for the selective capture and identification of MDA-MB-231 TNBC cells and HER-2(+) breast cancer cells simultaneously from infected blood and cell mixtures, 10^5^ cells per mL of HER-2(−) ER(−) MDA-MB-231 cells, 10^5^ cells per mL of HER-2(+) SK-BR-3 cells and 10^5^ cells per mL of ER(+) MCF-7 cells were incubated with 10 mL citrated whole rabbit blood. In the next step, we added 10^6^ cells per mL peripheral blood mononuclear cells (PBMCs) to the mixture and subjected it to gentle shaking for more than 90 min. After that, different concentrations of an anti-HER-2 antibody attached to a blue fluorescent magnetic LCD-based nanosystem and an anti-AXL antibody attached to a green fluorescent magnetic LCD-based nanosystem were added to the infected blood sample and mixed continuously for 40 min. After that, the targeted cells bound to the anti-HER-2 antibody attached to the blue fluorescent magnetic LCD-based nanosystem and the targeted cells bound to the anti-AXL antibody attached to the green fluorescent magnetic LCD-based nanosystem were separated using a bar magnet. In the next step, the targeted cells bound to the anti-HER-2 antibody attached to the green fluorescent magnetic LCD-based nanosystem and the anti-AXL antibody attached to the green fluorescent magnetic LCD-based nanosystem were characterized using an ELISA kit and fluorescence mapping analysis, as shown in Fig. 6. As shown in Fig. 6C, ELISA experimental data show that the capture efficiency for HER-2(+) SK-BR-3 cells was greater than 98% and that for MDA-MB-231 TNBC cells was greater than 97% when we used the mixture of an anti-HER-2 antibody attached to a blue fluorescent magnetic LCD-based nanosystem and an anti-AXL antibody attached to a green fluorescent magnetic LCD-based nanosystem. On the other hand, the capture efficiency for ER(+) MCF-7 cells was less than 2%. Fig. 6B shows a multicolor blue/green single-photon luminescence image of magnetically captured cells, which indicates that HER-2(+) SK-BR-3 cells and MDA-MB-231 TNBC cells were captured simultaneously by the anti-HER-2 antibody attached to a blue fluorescent magnetic LCD-based nanosystem and the anti-AXL antibody attached to a green fluorescent magnetic LCD-based nanosystem. All the reported data clearly indicate that the anti-HER-2 antibody attached to a blue fluorescent magnetic LCD-based nanosystem and the anti-AXL antibody attached to a green fluorescent magnetic LCD-based nanosystem can be used in combination for the simultaneous separation and imaging of HER-2(+) SK-BR-3 cells and MDA-MB-231 TNBC cells.

## Conclusion

In conclusion, the current article reports the design of highly crystalline antibody-conjugated multifunctional multicolor-luminescence nanosystems derived from the naturally available popular tropical fruits mangoes and prunes. Our design of a bioconjugated multifunctional nanoprobe exhibits excellent magnetic and multicolor fluorescence properties for the selective separation and accurate identification of TNBC and HER-2(+) or ER/PR(+) breast cancer cells selectively and simultaneously. We have shown that by changing the fruits multicolor LCDs can be developed, which is mainly due to the presence of different types of surface functional group on the surface of the LCDs. We have also reported that with a change of fruits the doping of different heavy metals such as Zn, Mg, Se, Cu, and Mn varies greatly between different LCDs. All the above factors introduce new energy levels for electronic transitions with comparable intensities. Reported experimental data demonstrated that the multifunctional multicolor nanosystems are capable of the selective and simultaneous capture of targeted TNBC, HER-2(+) or ER(+) breast cancer cells, and the capture efficiency can be as high as 98%. We have also shown that the multicolor nanosystems are capable of mapping heterogeneous breast cancer cells simultaneously and can distinguish targeted TNBC cells from non-targeted HER-2(+) or ER/PR(+) breast cancer cells. Our findings indicate that the fruit-based green approach has excellent potential for the design of multicolor nanosystems for detecting cancer heterogeneity in clinical practice.

## Experimental

All fruits were purchased from the local market. All the chemicals, including H_3_PO_4_, ethanol, CH_2_Cl_2_, NH_2_-PEG, FeCl_3_·6H_2_O, and 1,6-hexanedioic acid, were purchased from Fisher Scientific and Sigma-Aldrich. The human triple-negative breast cancer (TNBC) cell line MDA-MB-232, the human HER-2(+) breast cancer cell line SK-BR-3, the human ER(+) and PR(+) breast cancer cell line MCF-7 and the HaCaT normal skin cell line were purchased from the American Type Culture Collection (ATCC, Rockville, MD).

### Development of blue fluorescent LCDs using prunes

Blue fluorescent LCDs were synthesized by the hydrothermal treatment of prunes using H_3_PO_4_. For this purpose, 0.5 g of dried prunes was dissolved in 5 mL of water and 10 mL of H_3_PO_4_. After sonication for 5 min, the solution was added to an autoclave and heated at 180 °C for 2 h. After that, the pH was adjusted to neutrality with NaOH. Finally, the product was dialyzed against water for 72 h (molecular weight cut-off [MWCO]: 1000 Da) and then filtered. The blue fluorescent carbon dots were further purified by dialysis and then stored at 4 °C. Yield: 0.4 g, 30%.

### Development of green fluorescent LCDs from mangoes

Green fluorescent LCDs were synthesized by the hydrothermal treatment of mangoes using H_3_PO_4_. The experimental details are described in the experimental section. In this case, we used a very similar experimental procedure to that used for prunes. For this purpose, 0.5 g of ripe mango was dissolved in 5 mL of water and 15 mL of H_3_PO_4_. After sonication for 5 min, the solution was added to an autoclave and heated at 200 °C for 2 h. After that, the pH was adjusted to neutrality with NaOH. Finally, the product was dialyzed against water for 72 h (molecular weight cut-off [MWCO]: 1000 Da) and then filtered. The green fluorescent carbon dots were further purified by dialysis and then stored at 4 °C. Yield: 0.5 g, 40%.

### Design of carboxylic acid-conjugated Fe_3_O_4_ nanoparticles

We designed carboxylic acid-functionalized Fe_3_O_4_ magnetic nanoparticles using a co-precipitation method from ferric chloride and 1,6-hexanedioic acid, as we reported previously,^16–19,29^ as shown in Scheme 1B. The experimental details have been reported previously.^16–19,29^ Finally, the Fe_3_O_4_ nanoparticles were separated from the supernatant using a magnet.

### Design of blue/green fluorescent magnetic LCD-based nanosystem

For the covalent attachment of blue/green fluorescent LCDs to acid-functionalized magnetic nanoparticles, EDC-mediated esterification was used, as we reported previously.^17,29^ After that, the final product, namely, ester-coupled blue/green fluorescent LCDs attached to multifunctional magnetic nanoparticles, was separated by a magnet and washed several times with water to remove excess LCDs.

### Development of antibody-conjugated fluorescent LCDs attached to magnetic nanosystems

For the selective separation and accurate identification of TNBC and HER-2(+) or ER/PR(+) breast cancer cells from blood, we have developed antibody-conjugated multifunctional multicolor-fluorescence magnetic LCD-based nanosystems. For this purpose, we developed amine-conjugated polyethylene glycol (NH_2_-PEG) attached to a blue/green fluorescent magnetic LCD-based nanosystem, and then a TNBC-targeted anti-AXL antibody was attached to a green fluorescent magnetic LCD-based nanosystem by our previously reported method.^16–19,29,46,49^ Similarly, for the selective separation and imaging of HER-2(+) SK-BR-3 cells we have developed an anti-HER-2 antibody attached to a blue fluorescent magnetic LCD-based nanosystem using the above procedure. On the other hand, for the selective separation and imaging of estrogen receptor (ER)(+) MCF-7 cells, an anti-ERa antibody was attached to a blue fluorescent magnetic LCD-based nanosystem using the above procedure.

### Cell culture and incubation with antibody-conjugated blue/green fluorescent multifunctional magnetic fruit-based LCD nanosystems

We purchased the triple-negative breast cancer (TNBC) cell line MDA-MB-232, the human HER-2(+) breast cancer cell line SK-BR-3, the human ER(+) and PR(+) breast cancer cell line MCF-7 and the HaCaT normal skin cell line, which were grown according to an ATCC procedure, as we reported previously.^16–19,29,46,49^ Once the cell culture count was greater than 10^6^ cells per mL, different concentrations of antibody-conjugated blue/green fluorescent multifunctional magnetic fruit-based LCD nanosystems were mixed with different cell lines for 30 min. After that, unbound antibody-conjugated blue/green fluorescent multifunctional magnetic fruit-based LCD nanosystems were separated using centrifugation followed by washing with buffer three times, to make sure that antibody-conjugated blue/green fluorescent multifunctional magnetic fruit-based LCD nanosystems that were not bound to cells were separated.

### Multicolor luminescence imaging of captured cancer cells

For the fluorescence imaging of antibody-conjugated blue/green fluorescent multifunctional magnetic fruit-based LCD nanosystems attached to TNBC and HER-2(+) or ER/PR(+) breast cancer cells, we used an Olympus IX71 inverted confocal fluorescence microscope fitted with a SPOT Insight digital camera, as we reported previously.^16–19,29,46,49^

### Cell viability assay

To study the cytotoxicity of the antibody-conjugated blue/green fluorescent multifunctional magnetic fruit-based LCD nanoprobes, different concentrations of cancer and normal cells, namely, TNBC and HER-2(+) or ER/PR(+) breast cancer cells and HaCaT cells, were treated with the nanosystems at different time intervals. Finally, we measured the cell viability using an MTT (3-(4,5-dimethylthiazol-2-yl)-2,5-diphenyltetrazolium bromide) assay using a Multiskan Ascent plate reader with Ascent software (Labsystems), as we reported previously.^16–19,29,46,49^

## Conflicts of interest

There are no conflicts to declare.

## Supplementary Material
